# Normothermic Versus Hypothermic Cardiopulmonary Bypass in Children Undergoing Open Heart Surgery (Thermic-2): Study Protocol for a Randomized Controlled Trial

**DOI:** 10.2196/resprot.4338

**Published:** 2015-05-25

**Authors:** Sarah Baos, Karen Sheehan, Lucy Culliford, Katie Pike, Lucy Ellis, Andrew J Parry, Serban Stoica, Mohamed T Ghorbel, Massimo Caputo, Chris A Rogers

**Affiliations:** ^1^Clinical Trials and Evaluation UnitSchool of Clinical SciencesUniversity of BristolBristolUnited Kingdom; ^2^Bristol Royal Hospital for ChildrenDivision of Women and ChildrenUniversity Hospitals Bristol NHS Foundation TrustBristolUnited Kingdom; ^3^Bristol Heart InstituteUniversity Hospitals Bristol NHS Foundation TrustBristolUnited Kingdom

**Keywords:** pediatrics, cardiac surgery, cardiopulmonary bypass, temperature, hypothermia, normothermia, clinical trials, randomized

## Abstract

**Background:**

During open heart surgery, patients are connected to a heart-lung bypass machine that pumps blood around the body (“perfusion”) while the heart is stopped. Typically the blood is cooled during this procedure (“hypothermia”) and warmed to normal body temperature once the operation has been completed. The main rationale for “whole body cooling” is to protect organs such as the brain, kidneys, lungs, and heart from injury during bypass by reducing the body’s metabolic rate and decreasing oxygen consumption. However, hypothermic perfusion also has disadvantages that can contribute toward an extended postoperative hospital stay. Research in adults and small randomized controlled trials in children suggest some benefits to keeping the blood at normal body temperature throughout surgery (“normothermia”). However, the two techniques have not been extensively compared in children.

**Objective:**

The Thermic-2 study will test the hypothesis that the whole body inflammatory response to the nonphysiological bypass and its detrimental effects on different organ functions may be attenuated by maintaining the body at 35°C-37°C (normothermic) rather than 28°C (hypothermic) during pediatric complex open heart surgery.

**Methods:**

This is a single-center, randomized controlled trial comparing the effectiveness and acceptability of normothermic versus hypothermic bypass in 141 children with congenital heart disease undergoing open heart surgery. Children having scheduled surgery to repair a heart defect not requiring deep hypothermic circulatory arrest represent the target study population. The co-primary clinical outcomes are duration of inotropic support, intubation time, and postoperative hospital stay. Secondary outcomes are in-hospital mortality and morbidity, blood loss and transfusion requirements, pre- and post-operative echocardiographic findings, routine blood gas and blood test results, renal function, cerebral function, regional oxygen saturation of blood in the cerebral cortex, assessment of genomic expression changes in cardiac tissue biopsies, and neuropsychological development.

**Results:**

A total of 141 patients have been successfully randomized over 2 years and 10 months and are now being followed-up for 1 year. Results will be published in 2015.

**Conclusions:**

We believe this to be the first large pragmatic study comparing clinical outcomes during normothermic versus hypothermic bypass in complex open heart surgery in children. It is expected that this work will provide important information to improve strategies of cardiopulmonary bypass perfusion and therefore decrease the inevitable organ damage that occurs during nonphysiological body perfusion.

**Trial Registration:**

ISRCTN Registry: ISRCTN93129502, http://www.isrctn.com/ISRCTN93129502 (Archived by WebCitation at http://www.webcitation.org/6Yf5VSyyG).

## Introduction

### Background

The treatment of many forms of congenital heart disease has continued to advance, and primary early repairs of an increasing number of defects are routinely performed (eg, atrioventricular canal, tetralogy of Fallot, transposition of the great arteries). At Bristol Royal Hospital for Children (BRHC) an average of 285 cardiac operations are performed a year, and approximately 75% (ie, an average of 210) require cardiopulmonary bypass (CPB). Despite its widespread use, there is still significant morbidity related to the nonphysiological nature of total CPB [1]. Whole body cooling (ie, hypothermia) is an integral part of congenital cardiac surgery, with most procedures being conducted between 28°C and 30°C, depending on the expected duration and type of operation. The main rationale for body cooling is to protect organs such as the brain, kidneys, and heart from ischemic injury by reducing the metabolic rate and, hence, oxygen consumption [1]. Nevertheless, hypothermia has a number of disadvantages, including detrimental effects on enzymatic function, energy generation, and cellular integrity [1]. Perfusion of the body and the brain at normal body temperature (ie, normothermia) is a potentially more physiological method to maintain the functional integrity of major organ systems, and in recent years there has been an increasing interest in normothermic CPB in adult and pediatric cardiac surgery [2-9]. The concept that normothermic systemic perfusion may confer certain advantages over hypothermic regimes arose fortuitously from adult clinical experience in which an absence of shivering, hemodynamic stability, minimum use of inotropes, and early extubation were observed when patients were not cooled [2]. This led several investigators to study the effects of systemic hypothermia and normothermic perfusion upon cellular and organ function [2,10-14].

In both adult and pediatric cardiac surgery, many of the detrimental effects of CPB on end organ dysfunction were previously believed to be mediated by activation of the inflammatory response [1,15]. One may expect that CPB-related systemic inflammatory response syndrome and multiorgan injury to be enhanced during normothermia, since most enzymatic processes occur optimally at 37°C. Supportive of this notion are clinical studies in adults [16] in which normothermic CPB (35°C-37°C) was associated with significantly elevated levels of inflammatory markers compared to hypothermic CPB (28°C-30°C). Consistent with this, previous animal data have shown that the inflammatory response is reduced by hypothermia [17,18]. However, a conflicting picture is emerging from research. Ohata et al [19] have demonstrated an attenuation of certain inflammatory mediators following warm systemic perfusion (34°C) compared to hypothermic perfusion (28°C). In a clinical study at the Bristol Heart Institute [10,20], normothermic (37°C) perfusion was also associated with attenuation of inflammatory mediator release in the postoperative period, compared to moderately hypothermic (32°C) and hypothermic CPB (28°C). In contrast, others have suggested that induction of a systemic response is not temperature dependent. Rasmussen et al have shown that the release of systemic inflammatory mediators after cardiac surgery in adults was independent of mild hypothermia (32°C) when compared to normothermia (36°C) [21]. A randomized controlled trial (RCT) in 66 children having open heart surgery, who were randomized to either moderate hypothermia (24°C) or mild hypothermia (34°C), found that neither the systemic inflammatory response nor organ injury were influenced by bypass temperature [22]. Eggum et al demonstrated that there were only minor differences in inflammatory marker concentrations between pediatric patients undergoing moderate (25°C) hypothermia and those with mild (32°C) hypothermia during CPB [23]. This evidence was supported by an RCT carried out at BRHC comparing warm (35°C-37°C) and cold (28°C) CPB on simple congenital cardiac malformations, which indicated that both whole body inflammatory response and myocardial reperfusion injury were similar between the 2 groups [24]. In addition, Caputo et al and Eggum et al demonstrated that normothermic CPB was associated with reduced oxidative stress compared with hypothermic CPB. Other researchers have studied the effect of the temperature of the cardioplegia during pediatric cardiac surgery [25,26]. Poncelet et al have taken this a step further by studying the effect of the temperature of the CPB in addition to the cardioplegia in an RCT on 47 children having cardiac surgery [27]. In this study, children were randomized to either mild hypothermia with cold crystalloid cardioplegia (CPB temperature 32°C, cardioplegia at 5°C) or normothermic with intermittent warm blood cardioplegia (CPB temperature 36.5°C). They found no significant difference in the cellular ischemic insult to the heart or in the early and late neurodevelopmental status of the patients. With conflicting evidence arising from clinical trials comprising relatively low numbers of patients and mainly simple cardiac surgery cases, it is difficult to establish whether the induction of a systemic response is temperature dependent and the impact this may have on clinical outcome. This warrants a larger study with clinical outcomes as primary endpoints.

### Aim

The Thermic-2 study (Current Controlled Trials ISRCTN93129502) will compare the clinical effectiveness of normothermic (35°C-37°C) versus hypothermic (28°C) CPB for the repair of common congenital cardiac pathologies. We will test the hypothesis that maintaining the body at 35°C-37°C (normothermia) rather than at 28°C (hypothermia) during pediatric open heart surgery reduces the whole body inflammatory response to the nonphysiological CPB and its detrimental effects on different organ functions, resulting in a better clinical outcome.

## Methods

### Study Design

**Figure 1:**
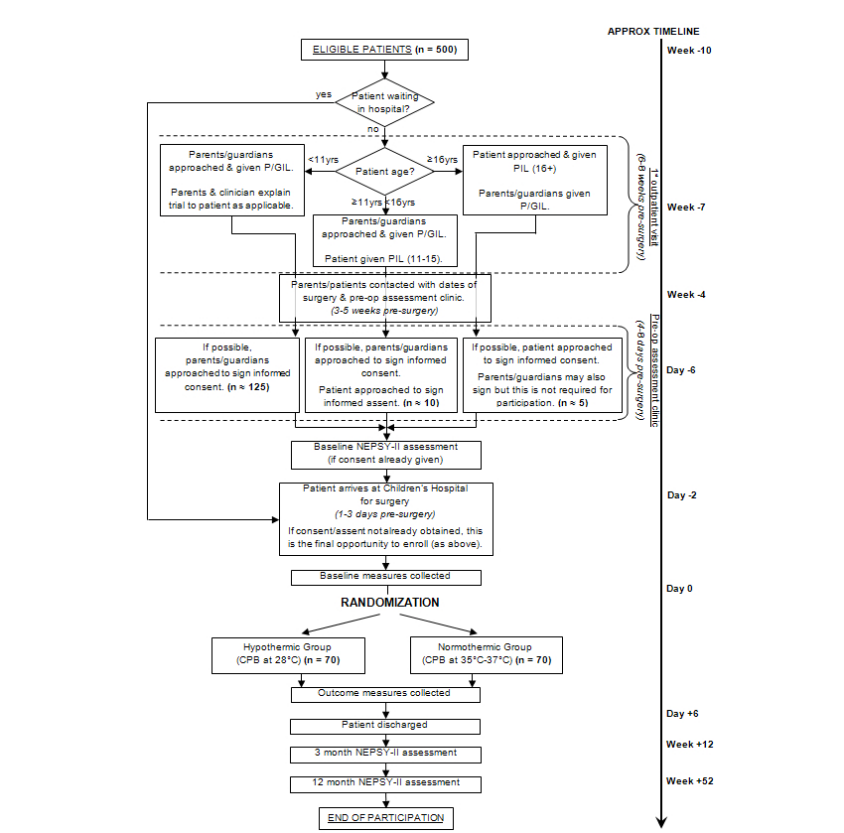
Study schema showing the participant recruitment pathway.

This study is a single-center, parallel-group open RCT. The study schema is shown in Figure 1.

### Research Approval

Research ethics approval was granted by the National Research Ethics Service Committee South West—Central Bristol (reference 11/SW/0122) in October 2011. The study is registered (ISRCTN 93129502).

### Study Population

All pediatric patients (aged 18 years and younger) having scheduled surgery at the BRHC to repair a congenital heart defect using CPB, represent the target study population and will be screened for eligibility. Patients will be excluded from the study if either: (1) they require deep hypothermic circulatory arrest, (2) they are admitted for an emergency operation (patients with hemodynamic instability who require immediate surgical intervention), or (3) they and/or their next-of-kin do not provide written informed consent. We will also record specific reasons where surgeons are unwilling for the patient to be approached for the study. All reasons for ineligibility will be recorded on the study screening log.

We will aim to approach all next-of-kin (usually parents) and/or patients at preoperative clinics or on admission the day before surgery. Eligible next-of-kin and/or patients will be given a patient information leaflet (PIL), have the study explained to them, and will be given the opportunity to ask questions about the study. They will be given a minimum of 24 hours to consider the study prior to being asked to provide written informed consent if they are willing to participate in the study.

### Randomization

Participants will be randomly assigned in a 1:1 ratio to either the hypothermic group or the normothermic group. Randomization will be stratified by age: 1 month or younger, 1 to 12 months, and older than 12 months. Allocations will be generated by computer using block randomization with varying block sizes. The allocation sequence will be prepared in advance of the study by a statistician independent of the study team. If a participant’s surgery is unexpectedly rescheduled, he/she will retain his/her study numbers and randomized allocation. Access to the allocation will be via a password-controlled secure database. Randomization will take place as close to the start of surgery as possible.

### Study Interventions

Participants will be randomized to receive either hypothermic or normothermic CPB during their surgery into the following groups:

1. Hypothermic CPB: cardiopulmonary bypass with perfusion at 28°C (control)
2. Normothermic CPB: cardiopulmonary bypass with perfusion at 35°C-37°C (experimental)

### Cardiopulmonary Bypass

**Figure 2:**
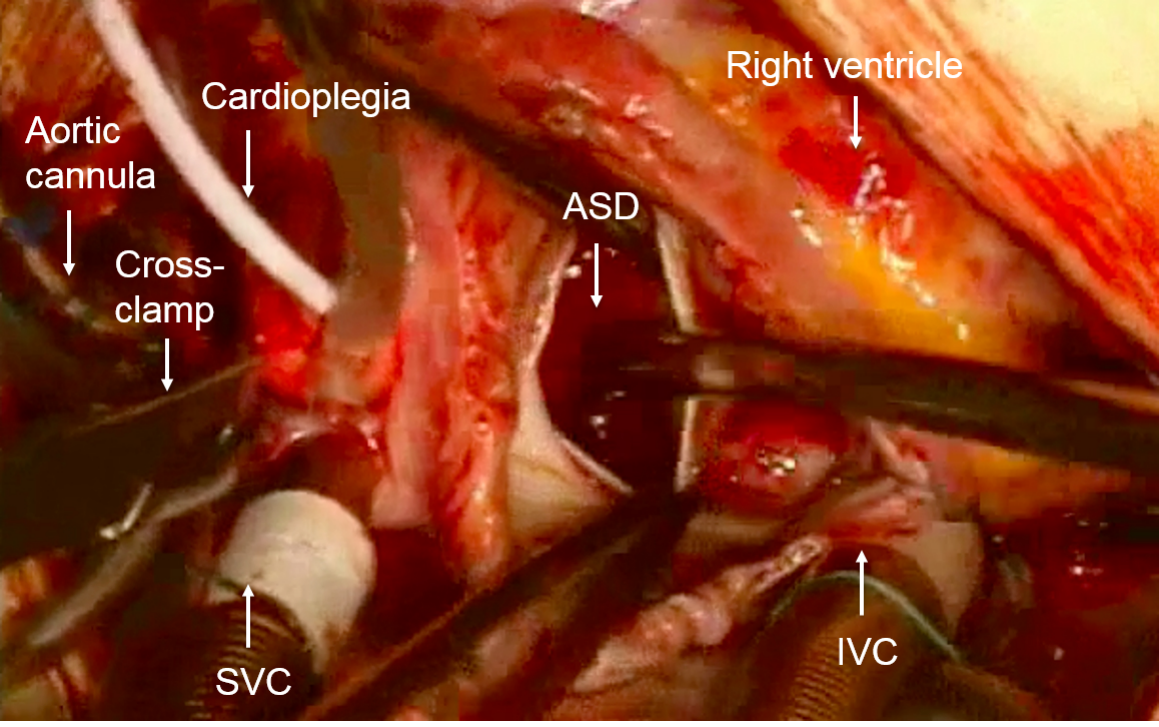
Image showing the heart of a child born with an atrial septal defect (ASD). The heart has been emptied of blood, put on cardiopulmonary bypass (CPB), and arrested using the cardioplegia solution, which is injected into the aortic root after the aorta is cross-clamped. CPB is achieved by inserting a superior vena cava (SVC) and an inferior vena cava (IVC) cannula for the venous drainage and an aortic cannula for the arterial perfusion of the body. The right atrium is open and the ASD is clearly visible.

All operations will be performed using CPB with ascending aortic cannulation and bicaval venous cannulation (Figure 2). Cold St Thomas’ I-based blood cardioplegic solution (4°C-6°C) will be used for myocardial preservation in all participants (Martindale Pharmaceuticals Ltd) with the following composition: 16 mM MgCl2, 2 mM CaCl2, 20 mM KCl, 147 mM NaCl, and 1 mM procaine HCl. Cardioplegic arrest will be achieved by an antegrade infusion of 110 ml/m^2^/min for 4 minutes. Additional cardioplegia will be administered after 20 minutes of aortic cross-clamping. Intramyocardial temperature will be monitored by means of a temperature probe inserted every 10 minutes into the right and left ventricles during ischemic arrest. For all participants, concomitant rectal, nasopharyngeal, skin, and blood temperatures will be monitored throughout the operation.

#### Hypothermic CPB

Nasopharyngeal body temperature will be lowered to 28°C. Rewarming will commence at the completion of the anatomical correction. All participants will be rewarmed with a temperature difference of 8°C at the level of the heat exchanger between the blood and the rewarming fluid, and CPB will be discontinued only after the participant is fully rewarmed to 36°C.

#### Normothermic CPB

Nasopharyngeal body temperature will be maintained at 35°C-37°C. Rewarming will only take place in the normothermic group if the body temperature is <36°C and will be discontinued only after the participant is fully rewarmed to 37°C.

### Anesthesia

Induction of anesthesia will be gaseous induction with sevoflurane, administration of neuromuscular blockade using pancuronium and analgesia using bolus dose fentanyl (10 mcg/kg-20 mcg/kg) pre-CPB, and morphine (0.5 mg/kg) during CPB. Maintenance of anesthesia will be with isoflurane. Alpha stat acid-base management will be adopted. Initial anticoagulation will be accomplished with 3 mg/kg to 4 mg/kg body weight of heparin and supplemented as required in order to maintain an active clotting time of ≥480 seconds.

Deviations from this standardized anesthetic technique are permissible under the following conditions:

1. Intravenous induction using ketamine, propofol, or thiopentone may be used if the anesthetist considers sevoflurane to be unsuitable for induction for the patient.
2. Where pancuronium is not available, vecuronium or rocuronium may be used instead.
3. Propofol instead of isoflurane may be used for maintenance of anesthesia where the anesthetist considers the patient to be at increased risk of awareness.

### Study Center and Surgeons

#### Overview

All surgery will be carried out at BRHC. On average, 210 pediatric cardiac surgery procedures using CPB are performed per year at this center. All the pediatric cardiac surgeons at this center are participating in the study.

#### Primary Outcomes

The co-primary endpoints are (1) duration of inotropic support, (2) intubation time, and (3) postoperative hospital stay (from date of surgery to discharge from cardiac ward). These data will be collected from medical notes and hospital charts.

#### Secondary outcomes

Data will be collected to characterize the following secondary outcomes:

1. In-hospital mortality and morbidity rates will be recorded.
2. Blood loss and transfusion requirements will be recorded.
3. Preoperative and postoperative echocardiographic (ECG) findings will be recorded.
4. Routine blood gas and blood test results will be recorded.
5. Renal function will be measured by testing urinary albumin, urinary creatinine, retinal binding protein (RBP), N-acetyl-β-glucosaminidase (NAG), and neutrophil gelatinase-associated lipocalin (NGAL) [28-33]. All markers of renal damage will be measured preoperatively and at several time-points postoperatively.
6. Cerebral function will be measured by testing for glial fibrillary acidic protein (GFAP), a serum marker of traumatic brain injury [34,35]. GFAP levels will be measured preoperatively and at several time-points mid- and postoperatively.
7. Regional oxygen saturation of blood in the cerebral cortex will be measured at approximately 15-minute intervals during the operative period using near-infrared spectroscopy (NIRS).
8. Neuropsychological development will be assessed using the NEPSY-II psychometric tool [36]. Neuropsychological assessments will be performed on patients aged between 3-16 years old, preoperatively and at 3 and 12 months postoperatively. These tests are intended as a tool to allow the assessment of both basic and complex aspects of cognition across the following 6 functional domains: attention and executive functions, language, memory and learning, sensorimotor functions, social perception, and visual-spatial processing.
9. Cardiac tissue biopsies from heart tissue discarded during the surgical procedure and considered clinical waste will be analyzed for biochemical markers and RNA analysis on a subset of 32 patients. If available, 4 samples will be collected from each patient in this subset; 2 samples will be collected immediately after institution of CPB and a further 2 samples will be collected 10-15 minutes after reperfusion. Biochemical tests will be performed on the biopsies to determine the presence of a range of important proteins and metabolites, and transcriptional profiling by RNA extraction will be performed with the aim to help establish whether any genomic expression changes associated with hypothermia could be prevented by using normothermic techniques.

### Assessment of Outcomes and Blinding

Participants and their parents/guardians will be blinded to the treatment allocation. Participants will receive the same surgical procedure as if they had not joined the study—with the exception of the temperature of the blood during CPB—and will receive the same surgical care following the procedure. At the start of the operation, the perfusion team is given the treatment allocation in a concealed envelope, asked to follow the study allocation, and record any reasons for protocol deviations. Surgeons, anesthetists, and nurses involved in the operation will be unblinded but the randomization allocation will not be disclosed until after the start of the operation. Because surgeons, anesthetists, and nurses continue to care for participants in the pediatric intensive care unit (PICU) and on the ward, it is difficult to maintain blinding in these locations. Where possible, staff will be blinded to the treatment group to which a participant is assigned. The PICU and ward nursing teams, while not being actively informed of the patient’s study allocation, could become unblinded because they have access to the anesthetic and perfusion charts that must be stored in the medical notes. However, it is unlikely that the nursing team would check the temperature at which the operation was performed or alter the patient’s after-care since the temperature during CPB would not have any implications on the postoperative management of the patient.

The primary endpoints of duration of inotropic support, intubation time, and postoperative hospital stay should all be objective outcomes and were chosen primarily as they are clinically meaningful to the patients and surgeons. While these outcomes could potentially be influenced by the clinical care team, the care team must not only adhere to strict protocols and guidelines, but there is also not a strong expectation that either arm of the study would be more beneficial to the participant; therefore, performance and detection bias are minimized.

Outcomes such as in-hospital mortality and morbidity, blood loss and transfusion requirements, routine blood gas, blood sample/test results, and regional oxygen saturation of blood in the cerebral cortex are objective outcomes and will be recorded directly from medical notes, PICU charts, and electronic records.

Preoperative and postoperative ECG findings involve some level of subjectivity and judgement; however, ECGs will be interpreted by a cardiologist blinded to treatment allocation. Renal function analysis, cerebral function analysis, cardiac tissue function analysis, and NEPSY-II test administration are not part of routine care, and assessment will be carried out by blinded researchers. Reasons for noncompletion of tests or assays will be recorded.

### Data and Sample Collection Schedule

The schedule for data and sample collection is shown in Table 1.

**Table 1:** Schedule of data and sample collection.

Data and samples collected	Preoperative	Perioperative				Postoperative									
		Pre-surgery	Start of CPB^a^	10m post CPB	XC^b^ removal	XC+ 30m^c^	XC+ 2h^d^	XC+ 4h	XC+ 6h	XC+ 24h	XC+ 48h	XC+ 72h	Discharge	3 mo^e^	12 mo
Eligibility	✓^f^														
Consent	✓														
NEPSY-II^g^	✓													✓	✓
Baseline data	✓														
Randomization		✓													
Routine blood gases		✓			✓	✓	✓		✓	✓					
Routine blood samples		✓					✓^h^			✓	✓	✓			
GFAP		✓			✓	✓	✓		✓	✓	✓				
NIRS		✓^i^													
Urine samples		✓			✓^j^			✓		✓	✓				
Cardiac tissue biopsies			✓	✓											
Operative details		✓	✓	✓	✓^k^										
Clinical outcomes													✓		
Safety data										✓	✓	✓	✓	✓^l^	✓

^a^CPB: cardiopulmonary bypass

^b^XC: cross clamp

^c^m: minutes

^d^h: hours

^e^mo: months

^f^✓ = data/sample collected

^g^Only performed on participants eligible for NEPSY-II psychometric assessment.

^h^Routine blood samples taken on admission to PICU.

^i^As many NIRS results will be recorded as are taken in theater.

^j^Urine samples taken at cross-clamp removal/end of CPB.

^k^Operative details are recorded on cross-clamp removal and chest closure.

^l^Hospital admission questionnaire only administered if 3-month NEPSY administered.

### Participant Follow-Up

All primary and most secondary outcomes are assessed while the participant is in hospital following their surgery. For NEPSY-II eligible patients, NEPSY assessments will take place at 3 months and 12 months postoperatively (Figure 1 and Table 1). Safety data will also be collected at these visits. For participants that are not eligible for NEPSY-II assessments, follow-up for safety will occur at 12 months postoperatively by postal questionnaire. Active participation for all patients will cease either at their final NEPSY-II assessment or on return of the follow-up postal questionnaire, 12 months post operatively.

### Sample Size

#### Primary Outcomes

The geometric mean postoperative hospital stay in our institution is estimated to be 6.2 days, with standard deviation (on the logarithmic scale) of 0.4. A sample size of 100 participants per group would be sufficient to allow us to detect a clinically relevant 1 day or greater reduction in mean length of stay with 90% power, assuming a 5% level of statistical significance (2-tailed).

Using estimates for ventilation time and duration of inotropic support from our institution, a total sample size of 200 would also be sufficient to detect a 16% reduction in ventilation time (3 hours), and a 13% reduction in inotropic support (4.7 hours). All these differences represent clinically relevant reductions.

In an earlier RCT comparing the same 2 interventions carried out at our institution (Thermic-1), 59 participants were recruited. The primary outcomes for this earlier study were biochemical markers of organ injury, but clinical outcome data were also collected. For the current study (Thermic-2), 141 participants have been recruited. The clinical data from the 2 studies will be combined for assessment of the clinical outcomes, giving a total sample size of 200 participants for analysis.

#### Secondary Outcomes

This sample size will also be able to detect clinically relevant differences in secondary outcomes with 90% power. For full details, see Multimedia Appendix 1.

### Statistical Analyses

#### Plan of Analysis

Binary outcomes will be compared using logistic regression. Quantitative outcomes will be transformed if necessary to achieve approximately normal distributions and compared using linear regression and time to event variables will be analyzed using survival methods. Outcomes with repeated measures (longitudinal data) will be analyzed using mixed models, which allow for unbalanced data. Alternative correlation structures will be considered, and the sensitivity of the results to the choice of structure examined. Analyses will be adjusted for age group (stratification factor). All analyses will be carried out on the basis of intention-to-treat. Outcomes will be reported as effect sizes with 95% confidence intervals.

Specific morbidities are too infrequent for the study to be able to detect statistically significant differences between groups. Frequencies of these adverse outcomes will be tabulated, in line with guidelines for reporting adverse events in trials, and reported in accordance with International Conference on Harmonisation Good Clinical Practice guidelines.

The analysis of the microarray data for the gene expression study—including normalization, noise reduction, and analysis using, for example, unsupervised hierarchical clustering and relevance network analysis—will be performed using Genespring 7 software. Following determination of the gene expression profiles, bioinformatics data mining tools (Genespring 7) will be used to identify the gene expression profiles that are differentially significant and to cluster them by biological function. This is vital for generating hypotheses about their role. Predefined stringent criteria will be used to select candidate genes for validation; only genes with statistically significant differential expression will be considered. Patterns of change in signaling pathway networks will be explored.

####  Subgroup Analyses

Effect estimates for the 2 study phases (Thermic-1 and Thermic-2) will be examined by adding relevant interaction terms to the models.

### Changes to the Protocol Since First Approved

After recruitment of 52 patients, an amendment was approved on August 24, 2012, which affected several secondary outcomes:

1. Markers of renal function were incorporated after new evidence emerged suggesting that normothermic CPB was associated with similar renal impairment to hypothermic CPB [31,33].
2. The marker for brain damage was changed from S100-B to GFAP and samples were taken over 48 hours rather than 24 hours. Evidence has suggested that, when measured systemically, GFAP is more specific to brain trauma and may take longer to return to baseline levels than S100-B levels, which may be elevated during an event such as CPB or when other organs are damaged or under stress and independently of whether trauma to the brain has occurred [37-42].
3. NIRS data collection was incorporated to provide insight into regional oxygen saturation of the blood in the cerebral cortex.

In addition, the list of expected adverse events was also updated to include junctional ectopic tachycardia and heart block. A second amendment was approved on September 3, 2013, in which the protocol was updated to clarify the end-of-study definition, enabling the distinction between the end of study for an individual participant and for the study as a whole.

## Results

A total of 141 patients have been successfully recruited over a 2-year, 10-month period for the Thermic-2 study. The participant follow-up period will end in October 2015 and results will be published in late 2015.

## Discussion

### Principal Findings

While whole body cooling is still very much an integral part of pediatric cardiac surgery in the belief that it provides some degree of protection against a systemic inflammatory response and multiorgan damage, there is little evidence to demonstrate that this translates to improved clinical outcomes [22-24,31,43]. This may, in part, be due to the multifactorial nature of CPB; the clinical outcome of a patient is likely to be affected by factors including the complexity of the anatomical defect being corrected and the time spent on CPB as well as the temperature of the CPB. For instance, correction of a simple anatomical defect performed using hypothermic CPB may attenuate inflammatory responses and organ damage, however, additional time spent on CPB to cool and rewarm the patient [22-24] may offset or mask these effects. In addition, most RCTs investigating normothermia versus hypothermia do not report the length of time spent at the allocated temperature, although through randomization the times should be similar in the 2 groups. Clinically, it is not appropriate to specify how long the target temperature must be maintained since CPB time should be kept to a minimum and rewarming must commence on completion of the anatomical correction. In practice, this results in shorter, less complex operations that potentially only receive the intervention for a short time period, while the longer operations may be exposed to the intervention for a greater length of time. Furthermore, we do not know how long the target temperatures of either 28°C or 35°C-37°C need to be maintained for physiological responses to take effect. While it would be difficult to impose a specific length of time for treatment intervention due to the constraints and complexity of surgery, it may be useful to collect information regarding the length of time during which the target temperature was maintained and this should be considered in future study design.

RCTs in children investigating CPB temperatures have recruited relatively low numbers of participants and have included the correction of primarily simple anatomical defects [23,24,27,43]. Furthermore, there is no agreed definition in the current literature of the reference temperature for hypothermic CPB; varying from as low as 24°C to up to 32°C, making interpretation of findings and cross-referencing difficult [8,22,23,27]. The Thermic-2 study has been designed to collect data from a large patient population with varying degrees of congenital heart defect complexity. The outcomes data have been chosen based on clinical relevance and with the aim to quantify postoperative course and multiorgan injury, focusing particularly on the heart, brain, kidney, and lungs. Also, these clinical parameters have been used to compare the 2 perfusion techniques in previous studies [8,9,22,27].

### Compliance

The study protocol clearly defines the target nasopharyngeal temperature during CPB to be either 35°C-37°C or 28°C. However, while the surgeon and theater team may be willing to follow the allocation, they could be faced with challenges in order to achieve strict adherence to the study temperature. The perfusion team responsible for controlling the temperature of the body throughout CPB are in charge of administering the allocation. While the perfusion equipment can be used to control temperature extremely accurately and can be set to hit an exact target temperature, the circumstances and nature of pediatric cardiac surgery can result in precise temperature control becoming a challenge; in practice, it takes time for the body temperature to change. For clinical reasons, the length of CPB should be kept to a minimum so the perfusionist must estimate how fast the body temperature will continue to drift down toward the target (eg, 28°C) prior to rewarming. This could result in either over- or under-shooting of the target temperature, and more often affects the hypothermic group, particularly on shorter cases, as the rewarming of the body is a clinically rate-limiting step, and rewarming may have to take place before the target temperature is reached. Additionally, an open chest, particularly on very small children, loses heat very quickly, and consequently the body temperature prior to starting CPB could drop and there may not be enough time to rewarm the patient in order reach the target temperature if they were allocated to the normothermic group (35°C-37°C). Finally, there may be circumstances when the surgeon may decide that, due to unforeseen circumstances, it is not clinically appropriate for the child to participate in the study. In this situation, the surgeon may dictate what temperature the CPB should be performed at and the reasons for noncompliance recorded.

### Minimization of Bias

The measures outlined below have been put in place to minimize potential bias: concealed randomization should prevent selection bias; blinding all possible staff, children, parents/guardians, and researchers will minimize performance and detection biases; the majority of outcomes are based on objective criteria; the PIL; and the process of obtaining informed consent will describe the uncertainty about the effects of normothermia versus hyperthermia and, therefore ,there should not be a strong expectation that one or the other method should lead to a more favorable result; attrition bias will be minimized by making every possible effort to keep in touch with participants; the study will be analyzed on an intention-to-treat basis, and every effort will be made to include all randomized patients.

### Conclusion

In summary, despite the challenges faced in delivering the temperature allocation during CPB, the study has proceeded successfully. Lessons learned from Thermic-2 should help to design and conduct future temperature-based congenital open heart surgery studies.

### Study Status

The study opened to recruitment in November 2011. Recruitment has recently completed, and follow-up of study participants continues.

## Multimedia Appendix 1

Sample size calculation for secondary outcomes.

## Abbreviations

ASD: atrial septal defect
BRHC: Bristol Royal Hospital for Children
CPB: cardiopulmonary bypass
ECG: electrocardiogram
GFAP: glial fibrillary acidic protein
IVC: inferior vena cava
NAG: N-acetyl-β-glucosaminidase
n-GAL: neutrophil gelatinase-associated lipocalin
NIRS: near infra-red spectroscopy
PICU: pediatric intensive care unit
PIL: patient information leaflet
RBP: retinal binding protein
RCT: randomized controlled trial
REC: research ethics committee
RNA: ribonucleic acid
SVC: superior vena cava
XC: cross clamp
