# Supplementary material for: Normothermic Versus Hypothermic Cardiopulmonary Bypass in Children Undergoing Open Heart Surgery (Thermic-2): Study Protocol for a Randomized Controlled Trial
Source: JMIR Res Protoc. 2015 May 25;4(2):e59. doi: 10.2196/resprot.4338 (PMC4460263; doi:10.2196/resprot.4338)
Supplement: Supplementary file 1 [file resprot_v4i2e59_app1.pdf]

Multimedia Appendix 1

Sample size calculation for secondary outcomes

This sample size will also be able to detect the following differences in secondary outcomes with 90% power:

i) Minimum of 0.29 standard deviations in GFAP biochemical marker of brain injury, assuming correlations of 0.7 between the pre- and post-measures and between the 5 post-measures (this outcome was not measured in Thermic-1).

ii) 0.25 standard deviations in serum creatinine and urea nitrogen, assuming correlations of 0.7 between the pre- and post-measures and between the 4 post-measures.

iii) 0.46 standard deviations in continuous measures such as blood loss.

iv) 0.33 standard deviations in neuropsychological scores, assuming correlations of 0.7 between the pre- and post-measures and between the 2 post-measures (this outcome was not measured in Thermic-1).

v) 0.32 standard deviations in renal function tests (with the exception of nGAL), assuming correlations of 0.7 between the pre- and post-measures and between the 4 post-measures (this outcome was measured in all patients in Thermic-1 but will only be measured in a subgroup of 60 Thermic-2 patients). 0.45 standard deviations in the nGAL renal function test, assuming correlations of 0.7 between the pre- and post-measures and between the 4 post-measures (this outcome was not measured in Thermic-1 and will only be measured in a subgroup of 60 Thermic-2 patients).
